# A scalable and durable polydimethylsiloxane-coated nanoporous polyethylene textile for daytime radiative cooling

**DOI:** 10.1515/nanoph-2023-0596

**Published:** 2023-11-10

**Authors:** Tong Wang, Xinyu Wu, Qian Zhu, Yinggang Chen, Shuqi Zhang, Min Gu, Yinan Zhang

**Affiliations:** Institute of Photonic Chips, University of Shanghai for Science and Technology, Shanghai 200093, China; Centre for Artificial-Intelligence Nanophotonics, School of Optical-Electrical and Computer Engineering, University of Shanghai for Science and Technology, Shanghai 200093, China

**Keywords:** daytime radiative cooling, bilayer structure, scalability, durability

## Abstract

Radiative cooling technology with zero-energy consumption and zero-carbon emission has drawn enormous attention. However, the high-cost manufacture, limited scalability, and narrow application scopes remain major impediments to radiative cooling commercialization. Here, we present a bilayer PDMS/nanoPE fabricated by an automatic film applicator for high-performance passive daytime radiative cooling. The nanoPE underlayer maximizes the reflection of sunlight and the transparent PDMS top-layer dramatically enhances the infrared emissivity of pristine nanoPE across the atmospheric transparency window (∆*E*_8–13 μm_ = 0.85). The obtained PDMS/nanoPE simultaneously allows a high solar reflectance of 0.94 and a thermal emittance of 0.94, enabling a sub-ambient cooling of 4.5 °C with a maximum of 7.6 °C in rooftop test and a theoretical net cooling power of 65 W/m^2^. A distinct temperature reduction of more than 10 °C can be achieved in comparison with pristine PDMS film. Integration of the hydrophobicity, durability, robust mechanical strength, and industrial scalability, we believe this work will provide practical and efficient solutions to cooling vehicles, buildings, and the human body in a simple and low-cost manner.

## 1 Introduction

According to an ongoing temperature analysis led by scientists at NASA’s Goddard Institute for Space Studies (GISS), the average global air temperature on Earth has increased by at least 1.1 °C since 1880 [1]. In recent decades, the growing demands for energy saving and heat mitigation concerns have drawn enormous attention to alternative cooling technology with zero-energy consumption and zero-carbon emission [2]. Radiative cooling for passive thermal management towards sustainable carbon neutrality instead of fossil fuels has become a pivotal subject, which is promising to replace or complement conventional cooling technologies [3]. Passive daytime radiative cooling (PDRC) simultaneously reflects sunlight (*λ* ∼ 0.3–2.5 μm) to minimize the solar heat gain and radiates heat through the atmospheric transparency window (*λ* ∼ 8–13 μm) to vastly harvest the coldness of the universe [4–10], harnessing the temperature difference between Earth’s surface at 275–300 K and outer space at 3 K [11].

Although previously demonstrated high-profile radiative coolers, such as multilayer photonic structures [8, 12–15], metamaterials [9, 16–18], porous polymers [10, 19–24], white cool-roof paints [25–30], and bioinspired structural materials [31–34], yield efficient daytime radiative cooling capabilities under direct sunlight, majority designs suffer from high prices and problems with scalability and applicability. Therefore, reducing the manufacturing cost, pursuing large-scale production methods [35, 36], and enhancing the cooling power remain key challenges [37–39]. Furthermore, radiative cooling textile has been recognized as one of the most attractive strategies owing to its significant potential in real-world applications, e.g., personal thermal management [18, 40, 41], vehicle cooling [42, 43], and building cooling [27, 44]. However, most radiative cooling textiles are neither designed for selective response in specific solar and mid-infrared (MIR) wavelengths nor feasible in industrial scale and cost-effective production.

In this work, we demonstrate a low-cost, scalable, and durable polydimethylsiloxane-coated nanoporous polyethylene textile (PDMS/nanoPE) for highly efficient passive daytime radiative cooling. The top-layer is designed as a transparent PDMS film that is one of the most prevalent polymers in PDRC and naturally ejects packets of infrared absorption/emission in the 8–13 μm range. The underlayer is a nanoPE that maximizes the reflection of the visible-near-infrared (Vis-NIR) band due to its multiple Mie resonances. This bilayer design provides an extended spectroscopic response, which concomitantly facilitates the reflection of the solar radiation (*R*_solar_ = 0.94), as well as, synergistically enhances the emittance across the atmospheric transparency window (*E*_8–13 μm_ = 0.94). This PDMS/nanoPE enables a comparable sub-ambient cooling of 4.5 °C with a maximum of 7.6 °C in rooftop test on a typical clear sunny day in Shanghai, China, and a theoretical net cooling power of 65 W/m^2^ under 1000 W/m^2^ of solar irradiance. Besides, the PDMS/nanoPE cooler simultaneously achieves several compelling attributes, such as hydrophobicity, durability, robust mechanical strength, high economic efficiency, and industrial applicability.

## 2 Results and discussion

### 2.1 Large-scale fabrication and characterization of PDMS/nanoPE

**Figure 1:**
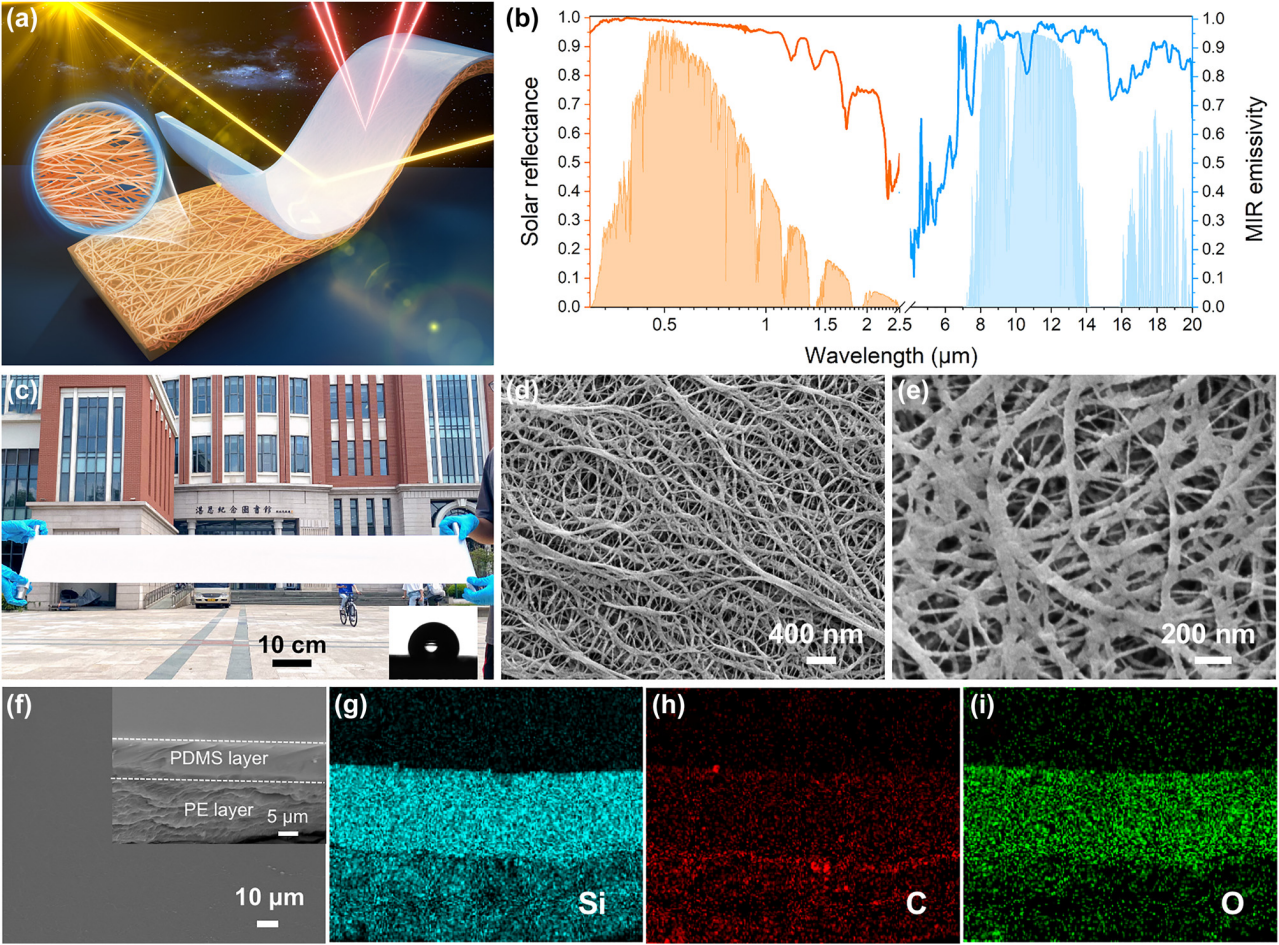
Optical properties and morphologies of PDMS/nanoPE. (a) The schematic of the proposed PDMS/nanoPE, consisting of transparent PDMS and nanoporous PE textile. (b) Solar reflectance and MIR emissivity of a 245-μm-thick of PDMS/nanoPE along with the normalized ASTM G173 Global solar spectrum and the LWIR atmospheric transparency window. (c) Photograph of the fabricated PDMS/nanoPE with the size of 100 cm × 20 cm, showing its bright white appearance and the scalable manufacturing. Inset: WCA of the PDMS/nanoPE surface. (d–e) SEM micrographs of the nanoporous PE textile with different magnifications. (f) SEM micrograph of PDMS/nanoPE top-layer. Inset: The cross-section of PDMS/nanoPE. (g–i) EDS elemental mappings of Si, C and O in the cross-section of PDMS/nanoPE.

As mentioned above, the ideal PDRC should possess high solar reflectance and strong thermal emittance. For this purpose, we propose a simple PDMS/nanoPE structure as illustrated in Figure 1(a), which consists of a 70-μm-thick transparent PDMS top-layer and a 175-μm-thick hierarchically nanoporous PE textile underlayer. The integrating bilayer design with 245 μm thickness presents a high average solar reflectance of 0.94 over the entire solar range and a high thermal emittance of 0.94 across the atmospheric transparency window (Figure 1(b)). Importantly, as shown in Figure 1(c), the PDMS/nanoPE enables a scale-up, low-cost and versatile manufacture and its top-layer displays enhanced hydrophobicity with a water contact angle (WCA) of 115° due to the lower surface energy of PDMS film. The hydrophobicity conducives to the removal of dust from the PDMS/nanoPE using water flow (Figure S1), showing typical self-cleaning property [45, 46]. Besides, the strong sunlight scattering gives our PDMS/nanoPE a matte and ultra-white appearance. It is worth noting that the fabricated nanoporous PE textile has interconnected nanopores with a size of 50–200 nm and nanofibers with a diameter of less than 200 nm (Figure 1(d)–(e), Figure S2). The hierarchical nanopores and nanofibers strongly scatter UV-Vis-NIR light owing to Mie scattering and make nanoPE opaque to human eyes. After casting with planar PDMS film using an automatic film applicator (see the details in materials and methods), the obtained PDMS/nanoPE displays a distinct bilayer structure, as indicated by the cross-section micrograph and EDS elemental mappings of Si, C, and O in Figure 1(f)–(i).

**Figure 2:**
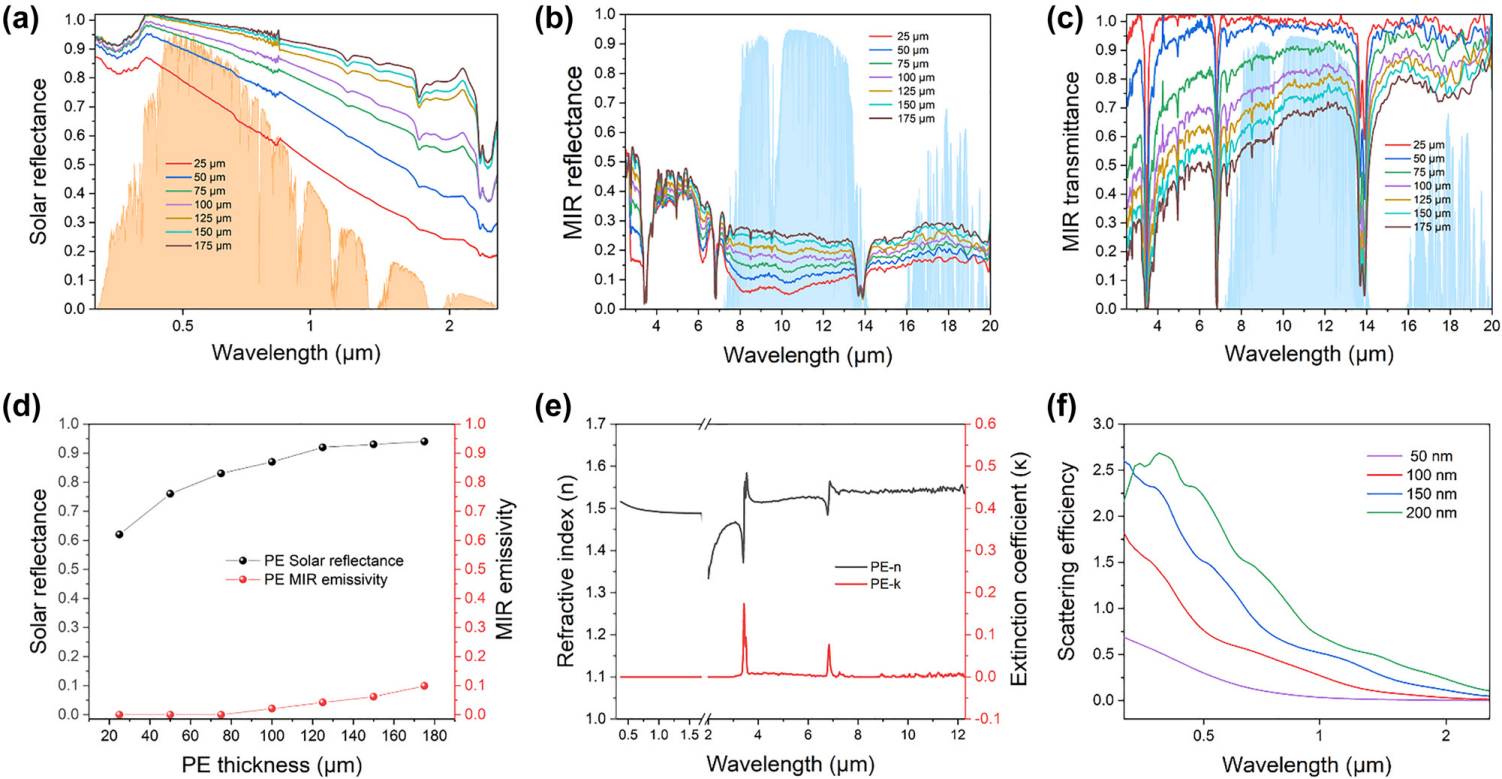
Experimental and simulated optical properties of the nanoPE. (a–c) Measured solar reflectance, MIR reflectance, and MIR transmittance spectra of the nanoPE. (d) Variations in the solar reflectance and MIR emissivity of the nanoPE with thickness. (e) Spectral refractive index (*n*) and extinction coefficient (*κ*) of PE. (f) Simulated scattering efficiency of the nanoPE with different diameters.

We further investigate the variations in the solar reflectance, MIR reflectance, and MIR transmittance spectra of the nanoPE with thickness (Figure 2(a)–(c)). As we can see in Figure 2(a), the average solar reflectance has a pronounced increasing trend with thickness and reaches to 0.94 when the thickness is 175 μm, which likely arises from the increased backscattering of light from the thicker, nonabsorptive, nanoporous PE textile. While the MIR reflectance appears to have no distinct change and the MIR transmittance decreases with increasing the nanoPE thickness (Figure 2(b)–(c)). According to Kirchhoff’s law, the emissivity through the atmospheric transparency window of 8–13 μm is calculated and maintains a low value of less than 0.1 due to the intrinsic characteristics of PE (Figure 2(d)). Clearly, the spectral refractive index (*n*) and extinction coefficient (*κ*) manifest the negligible absorptivity in the solar range and 8–13 μm wavelengths with the results shown in Figure 2(e), keeping the minimized heat gain from sunlight and a small amount of infrared absorption/emission in the atmospheric transparency window. To reveal the influence of PE fiber diameter on the scattering efficiency, finite-difference time-domain (FDTD) simulations were carried out as shown in Figure 2(f). Given the simulation results, one could conclude that various diameters of PE fiber collectively provide a broad-spectrum scattering efficiency across the visible wavelengths due to the high-order Mie resonance excitations.

**Figure 3:**
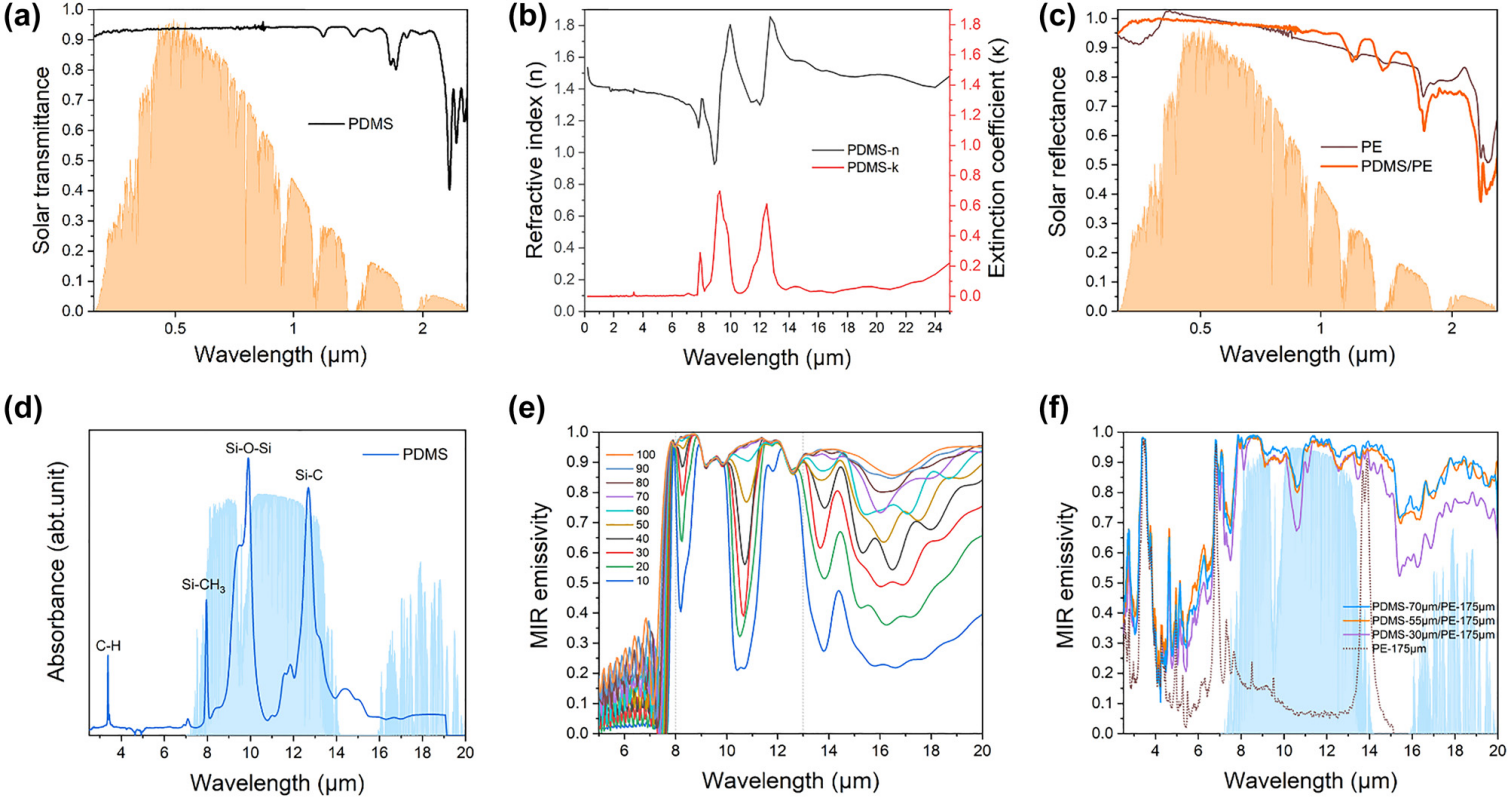
Experimental and simulated optical properties of the PDMS film and PDMS/nanoPE. (a) Measured solar transmittance spectrum of the PDMS film. (b) Spectral refractive index (*n*) and extinction coefficient (*κ*) of the PDMS film. (c) Solar reflectance comparison of the nanoPE and PDMS/nanoPE. (d) Absorbance spectrum of the PDMS film measured with ATR-FTIR spectroscopy. (e) Simulated the MIR emissivity of the PDMS film at different thicknesses. (f) Measured MIR emissivity of the PDMS/nanoPE at different PDMS thicknesses.

As one of the commercially available and inexpensive polymers, the PDMS is used to coat the nanoPE surface using an automatic film applicator. First, the free-standing PDMS film with thickness ∼300 μm is highly transparent in the visible light range and has a negligible extinction coefficient that can avoid extra heat gain from sunlight (Figure 3(a) and (b)). The solar reflectance spectra of the nanoPE and PDMS/nanoPE are compared in Figure 3(c) and both present the same high average solar reflectance of 0.94, further evidencing that the solar reflectance of nanoPE is insusceptible to vary with the addition of PDMS top-layer.

Secondly, the PDMS film has indispensable spectral characteristics across the atmospheric transparency window. It is observed in Figure 3(b)–(d) that PDMS film has multiple extinction peaks at 7.9, 9.3, and 12.5 μm because the Si–O–Si and Si–C chemical bonds and their various vibration modes, naturally ejecting packets of infrared absorption/emission in the infrared 8–13 μm range. Notably, the simulated emissivity of the PDMS film in 8–13 μm demonstrates an obvious increase in thickness and keeps an invariant value of 0.94 when the thickness is higher than 70 μm (Figure 3(e)). The measured MIR emissivity spectra of the PDMS/nanoPE at different PDMS thicknesses agree with the theoretical expectations and sought to achieve a dramatic emissivity promotion (∆*E*_8–13 μm_ = 0.85) in contrast to pristine nanoPE (Figure 3(f)). Intriguingly, the synergetic tunability of solar reflectance and MIR emissivity enables our PDMS/nanoPE to adapt to various requirements of different climates and avoid overcooling on winter days.

### 2.2 Subambient cooling performance of the PDMS/nanoPE radiative cooler

**Figure 4:**
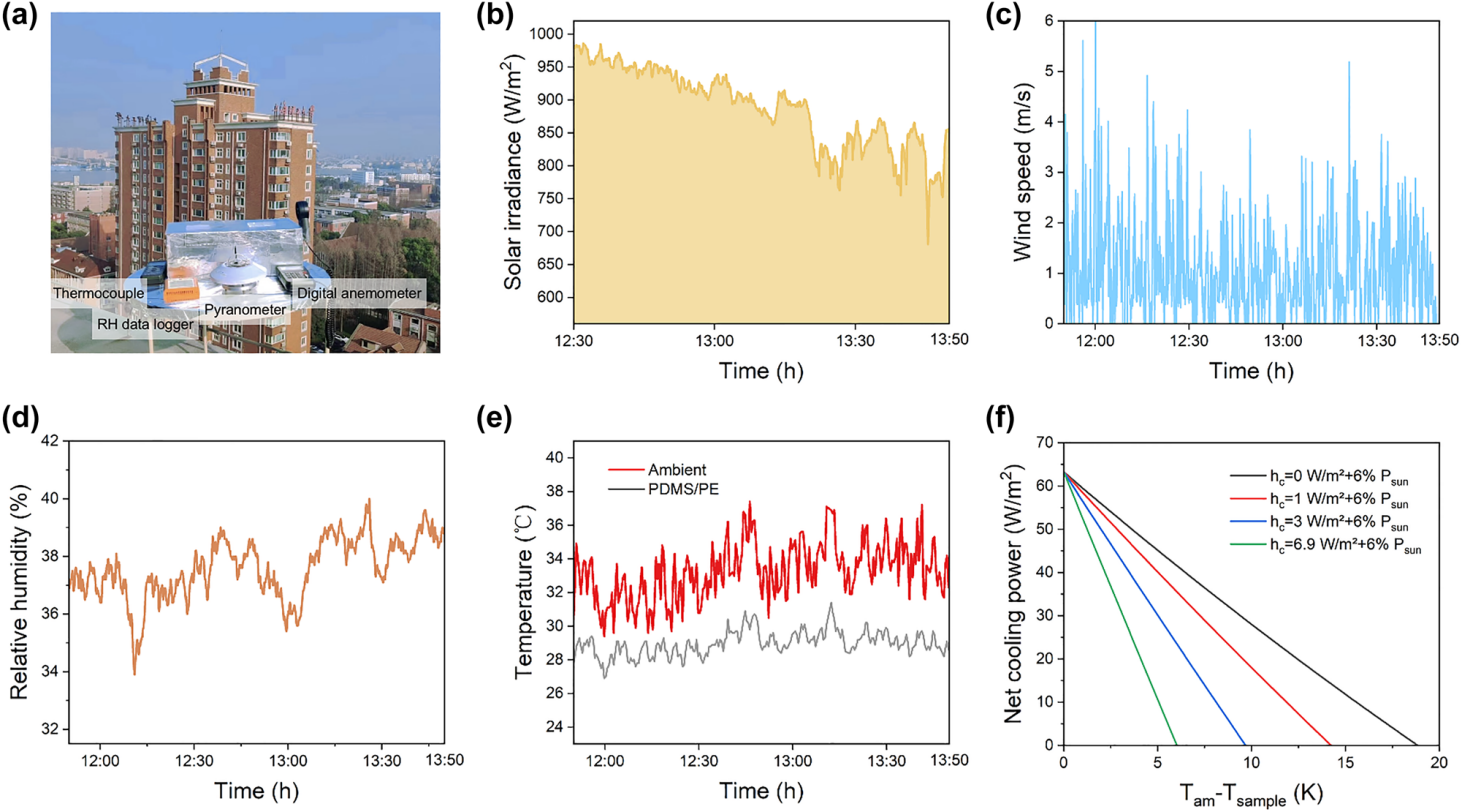
Experimental setup and subambient radiative cooling performance of PDMS/nanoPE. (a) Photo of the thermal measurement platform set up on the rooftop at the University of Shanghai for Science and Technology under a clear sky. (b–e) Measured solar irradiance, wind speed, relative humidity and the air and PDMS/nanoPE temperatures. (f) Calculated net cooling power of PDMS/nanoPE during the daytime. Heat transfer coefficient values of 0, 1, 3, and 6.9 W/(m^2^ K) are used in the calculations.

To experimentally test the radiative cooling performance of the PDMS/nanoPE, we set up a thermal measurement platform as shown in Figure 4(a). One can see the schematic diagram of the thermal platform in Figure S3, where the PDMS/nanoPE sample is placed inside heat-insulated foam to minimize the non-radiative heat exchange. Reflective aluminum foil is used to block solar irradiation and surrounding thermal emission. Under a solar irradiance of 890 W/m^2^, wind speed of 1.0 m/s, and relative humidity of 37 % in Shanghai city (Eastern China, Coastal, 31° 18′ 22″ N, 121° 30′ 17″ E), the temperatures of the air and PDMS/nanoPE were tracked in real-time at noon and the average temperature reduction of 4.5 °C with a maximum reduction of 7.6 °C is demonstrated (Figure 4(b–e)). To further elucidate the radiative cooling performance of the PDMS/nanoPE, we calculated the net cooling power as a function of the temperature reduction based on the experimental measured solar and emission spectra, demonstrating a net cooling power of 65 W/m^2^ (Figure 4(f)).

**Figure 5:**
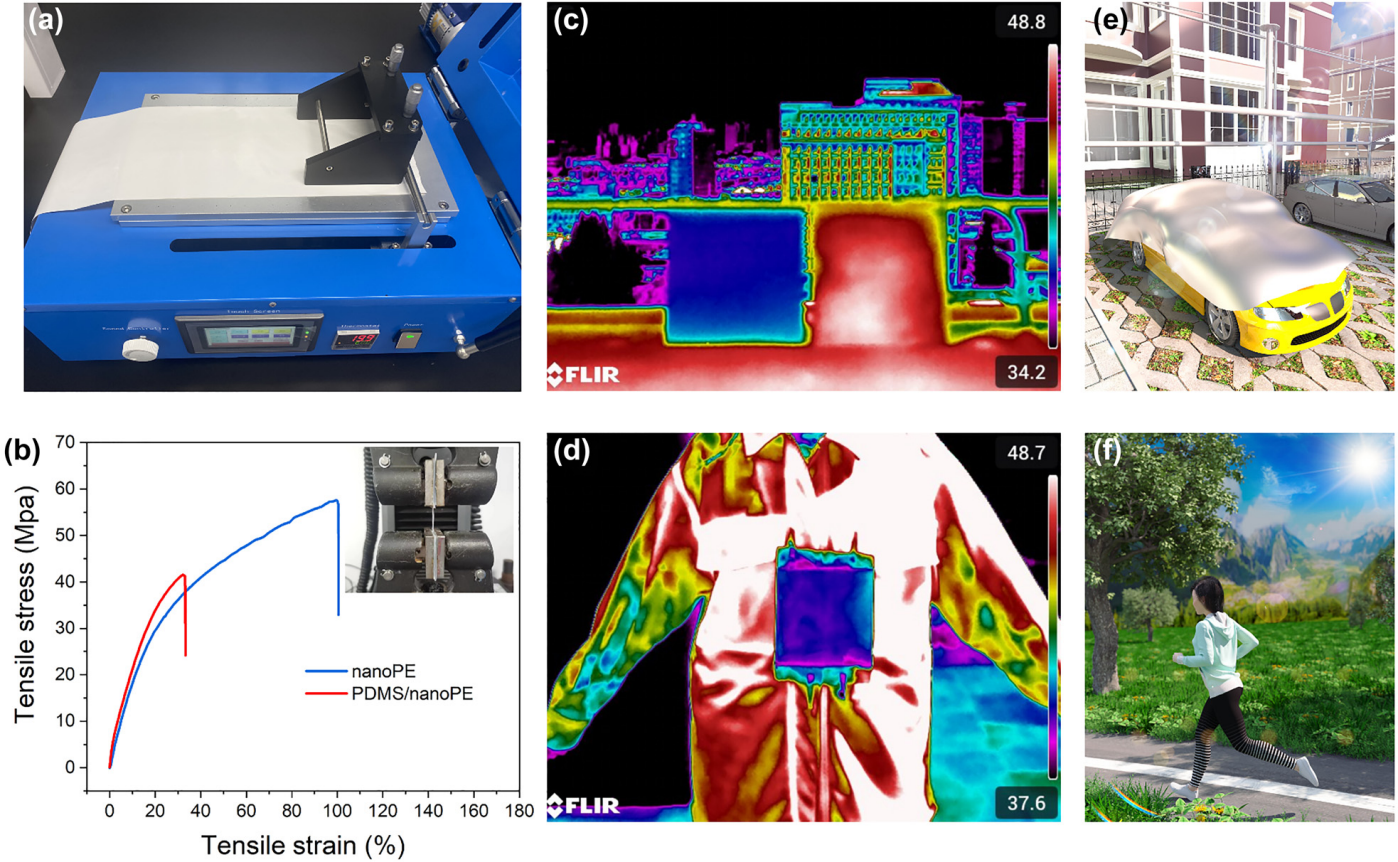
Manufacture and applications of the PDMS/nanoPE. (a) Photo of the fabrication process of the PDMS/nanoPE. (b) Mechanical strength tests of the nanoPE and PDMS/nanoPE versus elongation. (c) IR image of the PDMS/nanoPE (left) and pristine PDMS (right) on 15 × 15 cm glass plates. (d) IR image of the volunteer wearing a piece of PDMS/nanoPE under direct sunlight. (e–f) Schematic of the practical scenarios of vehicle and personal thermal management.

Promisingly, as we can see in Figure 5(a), our PDMS/nanoPE is manufactured via an automatic film applicator, which is an industrial scale and cost-effective production strategy. We further conducted the accelerated weathering testing for the PDMS/nanoPE under exposure to a variety of stimuli, such as heat, water, oxygen, and UV radiation. As shown in Table S1, even after accelerated weathering treatment for 30 days, the solar reflectance, thermal emittance, and water contact angle only show slight fluctuations and negligible variations with the accelerated weathering time. The PDMS/nanoPE displays excellent durability without any peeling, cracking, blistering, or discoloration. In addition, the nanoPE and PDMS/nanoPE both exhibit good tensile performance with an elongation of 100 % and 33 %, and breakage strength of 57 Mpa and 41 Mpa, respectively (Figure 5(b)). More exhilaratingly, from the IR images under direct sunlight in Figure 5(c and d), we can see a distinct temperature difference of more than 10 °C between the PDMS/nanoPE and pristine PDMS as well as human body, making the PDMS/nanoPE cooler attractive for practical scenarios, such as vehicle and personal thermal management (Figure 5(e and f)).

## 3 Conclusions

In summary, we demonstrate a low-cost, scalable, and durable PDMS/nanoPE fabricated by an automatic film applicator for high-performance passive daytime radiative cooling. This bilayer-structured PDMS/nanoPE design realizes a high solar reflectance of 0.94 and a thermal emittance of 0.94 across the atmospheric transparency window by controlling the PDMS viscosity, nanoPE thickness and coating speed. Furthermore, the PDMS/nanoPE enables a sub-ambient cooling of 4.5 °C with a maximum of 7.6 °C in rooftop test and a theoretical net cooling power of 65 W/m^2^ under 1000 W/m^2^ of solar irradiance. In comparison with pristine PDMS film, a distinct temperature reduction of more than 10 °C can be achieved under direct sunlight. Combining the hydrophobicity, durability, robust mechanical strength, and industrial scalability of the PDMS/nanoPE, we believe this work can bring vast opportunities for the next generation of PDRC applications.

## 4 Materials and methods

### 4.1 Fabrication

The nanoporous PE textile was fabricated via a phase-inversion-based method. In brief, PE powder, paraffin oil, butylated hydroxyto-luene (Sigma), and other additives were stirred at 150 °C for 3 h to produce a homogenous solution. The heated solution was pushed through a sheet die to make a gel-like film and stretched uniaxially. Then the as-formed gel was extracted with cyclohexane several times and dried to obtain the nanoporous PE textile. Next, the PDMS prepolymer (Sylgard 184 Silicone Elastomer), curing agent and toluene (10:1:10 mass ratio) were mixed and then cast onto the nanoporous PE textile using an automatic film applicator and subsequently cured at 80 °C for at least 1 h to prepare the PDMS/nanoPE.

### 4.2 Theoretical model of the radiative cooling performance

The net cooling power Pcool based on a thermal equilibrium equation can be expressed as:

(1) $$P_{\text{cool}}\left(T\right)=P_{\text{rad}}\left(T\right)-P_{\text{atm}}\left(T_{\text{amb}}\right)-P_{\text{Solar}}-P_{\text{cond }+\text{ conv}}$$

where *T* is the surface temperature and *T*_amb_ is the ambient temperature. The definitions of the thermal radiation power *P*_rad_(*T*), atmospheric radiation power *P*_atm_(*T*_amb_) and absorbed power from the incident solar irradiance *P*_Solar_ are given, respectively, as follows:

(2) $$P_{\text{rad}}\left(T\right)=A\int d\Omega \cos\theta \int_{0}^{\infty }d\lambda I_{BB}\left(T,\lambda \right)\varepsilon \left(\lambda ,\theta \right)$$

(3) $$\begin{array}{ll} P_{\text{atm}}\left(T_{\text{amb}}\right) & =A\int d\Omega \cos\theta \int_{0}^{\infty }d\lambda I_{BB}\left(T_{\text{amb}},\lambda \right)\varepsilon \left(\lambda ,\theta \right)\varepsilon_{\text{atm}}\left(\lambda ,\theta \right) \end{array}$$

(4) $$P_{\text{Solar}}=A\int_{0}^{\infty }d\lambda \varepsilon \left(\lambda ,\theta_{\text{Solar}}\right)I_{\text{AM}1.5}\left(\lambda \right)$$

Here, *A* is the area of the sky facing the cooler, and $\int d\Omega =2\pi \int_{0}^{\pi /2}d\theta \sin\theta$ is the angular integral over a hemisphere. $I_{BB}\left(T,\lambda \right)=\frac{2hc^{2}}{\lambda^{5}}\frac{1}{e^{hc/\left(\lambda \kappa_{B}T\right)}-1}$ is the spectral radiance of a blackbody at temperature *T*, *h* is Planck’s constant, *κB* is the Boltzmann constant, and *c* is the speed of light. *ɛ*(*λ*, *θ*) is the directional emissivity of the surface at wavelength *λ*. In equation (5), *ɛ*_atm_(*λ*, *θ*) = 1 − *τ*(*λ*)^1/con*θ*^ is the angle-dependent emissivity of the atmosphere, and *τ*(*λ*) is the atmospheric transmittance in the zenith direction according to the mid-latitude summer atmosphere model. *I*_AM1.5_ is the solar illumination spectra with an air mass of 1.5. *P*_cond+conv_ is the parasitic heat delivered to the cooler from the surrounding atmosphere, which can be evaluated according to the heat transfer equation as follows:

(5) $$P_{\text{cond }+\text{ conv}}\left(T,T_{\text{amb}}\right)=Ah_{c}\left(T_{\text{amb}}-T\right)$$

where *T*_amb_ − *T* is the temperature difference between the environment and the cooler surface. *h*_*c*_ = *h*_cond_ + *h*_conv_ is a combined nonradiative heat transfer coefficient considering both conductive and convective heat transfer due to the contact of PSHFHP with external air. The convection coefficient *h*_conv_ is estimated from an empirical equation *h*_conv_ = 5.7 + 3.8 V, where *V* is the experimentally measured average wind speed (*V*).

### 4.3 Numerical simulations

We performed finite-difference time-domain (FDTD) simulations by FDTD Solutions software (V8.19.1584, Lumerical Co. Ltd) to investigate the optical properties. For the nanoPE scattering calculation, a total-field scattered field was used to illuminate the fiber. The simulation boundaries were set as perfectly matched layer (PML) conditions to avoid the optical interference between the reflected light from the boundaries and scattered light. For the MIR emissivity simulation, a plane wave light source with a wavelength range of 5–20 µm was used to illuminate the PDMS. A PML condition was used as the bottom boundary of the simulation box to simulate an infinitely thick case.

### 4.4 Rooftop test

The radiative cooling performance of PDMS/nanoPE was investigated under a clear sky in Shanghai City (eastern China, coastal, 31° 18′ 22″ N, 121° 30′ 17″ E). A thermal box was designed with insulation foam covered by a layer of reflective foil to minimize both conductive and convective heat exchange. A relative humidity (RH) data logger (GSP-8, Elitech, Corp, China) with an accuracy of ±0.1 % RH was placed near the thermal box to measure the relative air humidity. The temperature detectors were mounted on the back surface of the films to detect real-time temperatures with an uncertainty of ±0.1 °C (CENTER309, CENTER Corp, Taiwan, China). The ambient temperature outside the box under direct sunlight was measured with the same thermometer. The solar irradiation incidence on the samples was simultaneously recorded using a data-logging solar radiometer with an accuracy of ±5 % (TES1333R, TES Electrical Electronic Corp. Taiwan, China). The wind speed around our thermal boxes was measured using a digital anemometer with an accuracy of ±2.5 % (AS856, Smart Sensor Corp, China). All weather data were automatically tracked every 10 s.

## Supplementary Material

Supplementary Material Details
