# Supplementary material for: A scalable and durable polydimethylsiloxane-coated nanoporous polyethylene textile for daytime radiative cooling
Source: Nanophotonics. 2023 Nov 10;13(5):601–9. doi: 10.1515/nanoph-2023-0596 (PMC11501767; doi:10.1515/nanoph-2023-0596)
Supplement: Supplementary file 1 — Supplementary Material Details [file j_nanoph-2023-0596_suppl_001.docx]

Supporting Information

A scalable and durable polydimethylsiloxane-coated nanoporous polyethylene textile for daytime radiative cooling

Tong Wang^1#^, Xinyu Wu^1,2#^, Qian Zhu^1,2^, Yinggang Chen^1,2^, Shuqi Zhang^1,2^, Min Gu^1^*, Yinan Zhang^1^*

^1^Institute of Photonic Chips, University of Shanghai for Science and Technology, Shanghai 200093, China.

^2^Centre for Artificial-Intelligence Nanophotonics, School of Optical-Electrical and Computer Engineering, University of Shanghai for Science and Technology, Shanghai 200093, China.

*Corresponding author. Emails: [zhangyinan@usst.edu.cn](mailto:zhangyinan@usst.edu.cn); [gumin@usst.edu.cn](mailto:gumin@usst.edu.cn)

**Characterizations**

The morphologies of nanoporous PE textile and PDMS/nanoPE were observed on a scanning electron microscope (Zeiss Gemini SEM 500) after gold coating, and the distribution of elements in the hybrid elastomer was examined by energy dispersive spectroscope (EDS) conducted on SEM. The optical reflectance and transmittance in the UV-Vis-NIR wavelength ranges were measured using a UV-Vis-NIR spectrophotometer (Hitachi, U-4100, Japan) equipped with a deuterium lamp for UV region, tungsten-halogen lamp for Vis, NIR range and a polytetrafluoroethylene integrating sphere. The thermal emission spectra in the mid-infrared wavelength ranges (2.5-20 μm) were characterized in an FTIR spectrometer (INVENIOR, Bruker) equipped with a deuterated triglycine sulfate crystal detector RT-DTGS, a gold integrating sphereA562 Integrating Sphere, Bruker and KBr beam splitter. Attenuated total reflectance-Fourier transform infrared spectroscopy (ATR-FTIR) (FTIR, Nicolet 6700, Thermo Fisher Scientific, USA) was carried out to reveal the infrared absorbance between 770 and 1250 cm-1 (8-13 µm) of the PDMS. Refractive index and extinction coefficient measurements in the wavelength range of 0.2-25 μm were taken for the PDMS film using a V-VASE and an IR-VASE ellipsometer (J. A. Woollam, USA).

The water contact angle (WCAs) of the PDMS/nanoPE was determined with an OCA15 contact angle analyzer (Dataphysics, Germany) to investigate the wetting property of the PSHFHP surface. The durability of the PDMS/nanoPE under long-term UV radiation was carried out in a QUV accelerated weathering tester (QUV/se, Q-Panel Co., Ltd, USA) with UV lamps at a wavelength of 310 nm. The PDMS/nanoPE samples were exposed to UV irradiation at 310 nm wavelength with intensity of 0.71 W/m2 at 60 °C for 4 h in dry conditions, followed by condensation at 50 °C for 4 h with UV lamps off. The thickness at five positions was measured via a digital display micrometer (Mintel, Germany) with a precision of ± 1 µm.

**Calculations of average solar reflectance and thermal emittance**

To accurately express the optical performance of the PDMS/nanoPE, we defined the average solar reflectance (R_solar_) as follows:

$R_{solar}=\frac{\int_{0.3\mu m}^{2.5\mu m} I_{solar}(\lambda)\cdot R_{solar}(\lambda, \theta)d\lambda}{\int_{0.3\mu m}^{2.5\mu m} I_{solar}(\lambda)\text{ d}\lambda}$ (1)

where λ is the wavelength of incident light in the range of 0.3-2.5 μm, $I_{solar}(\lambda)$ is the normalized ASTM G173 global solar intensity spectrum, and $R_{solar}(\lambda，\theta)$ is the surface’s angular spectral reflectance.

Similarly, the average thermal emittance (E_8-13μm_) in the atmospheric transparency window is defined as:

$E_{8-13\mu m}=\frac{\int_{8\mu m}^{13\mu m} I_{BB}(\lambda)\cdot\varepsilon_{LWIR}(\lambda, \theta)d\lambda}{\int_{8\mu m}^{13\mu m} I_{BB}(\lambda)\text{ d}\lambda}$ (2)

where $I_{BB}(\lambda)$ is the spectral intensity emitted by a blackbody and $\varepsilon_{LWIR}(\lambda, \theta)$ is the surface’s angular spectral thermal emittance in the range of 8-13 μm.

**
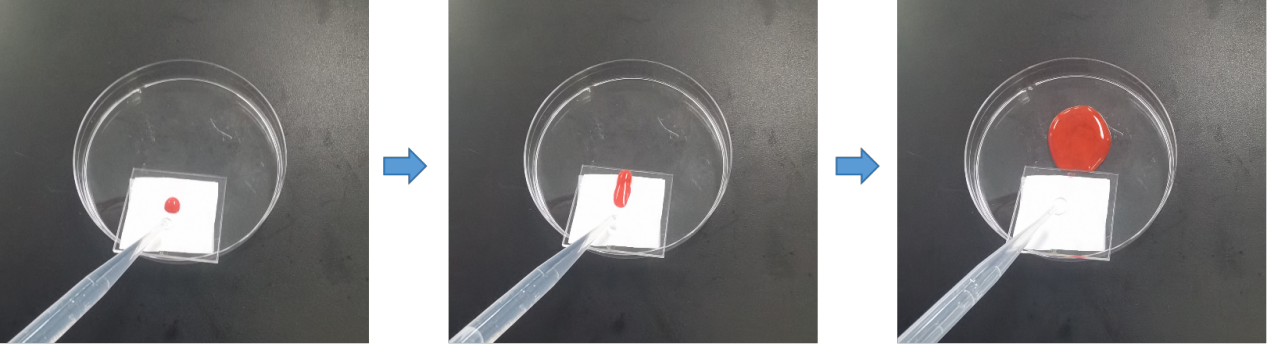
**

**Figure S1.** Dust self-cleaning test of the PDMS/nanoPE.

**
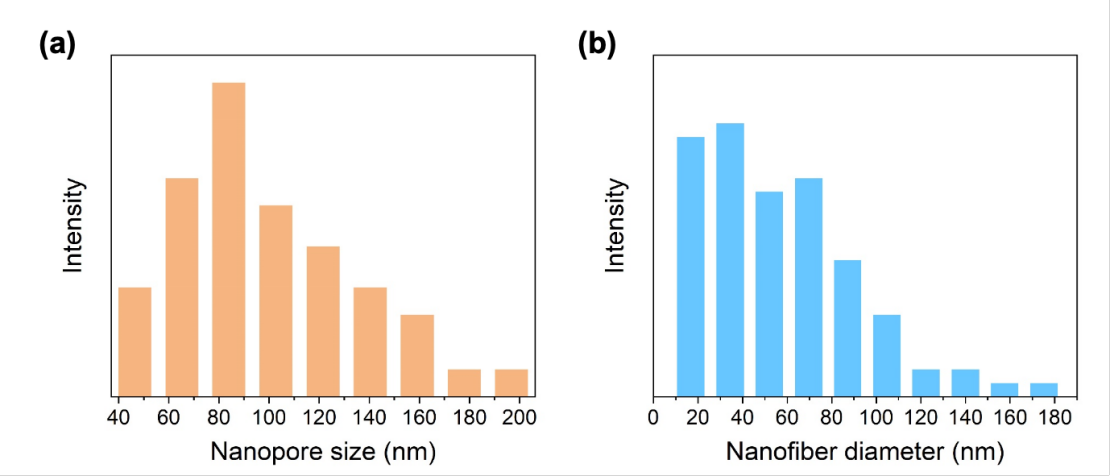
**

**Figure S2.** Nanopore size and nanofiber diameter distributions of polyethylene textile.

**
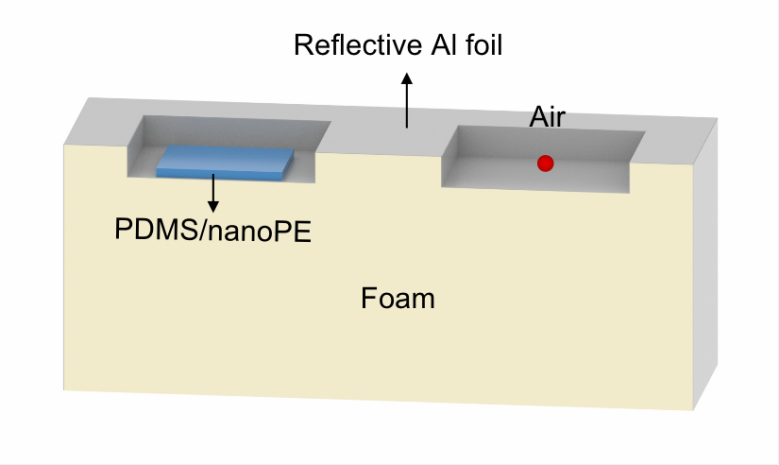
**

**Figure S3.** Schematic of the temperature measurement apparatus.

**Table S1.** Results of the accelerated weathering tests of the PDMS/nanoPE.

| **Days** | **Solar Reflectance** | **Thermal emittance** | **WCA (°)** |
| --- | --- | --- | --- |
| 10 | 0.943 | 0.942 | 116 |
| 20 | 0.936 | 0.944 | 113 |
| 30 | 0.938 | 0.936 | 114 |
